# Diagnostic efficacy of VELscope in oral potentially malignant disorders: a systematic review and meta-analysis

**DOI:** 10.3389/froh.2026.1811909

**Published:** 2026-04-17

**Authors:** Yan Li, Zhibang Li, Wenru Wang, Kun Li

**Affiliations:** 1 Department of Pharmacy, Jinan Stomatological Hospital, Jinan, China; 2 Department of Periodontology/Biomaterials, School and Hospital of Stomatology, Cheeloo College of Medicine, Shandong University, Jinan, China; 3 Shandong Key Laboratory of Oral Tissue Regeneration, Shandong University, Jinan, China; 4 Shandong Engineering Research Center of Dental Materials and Oral Tissue Regeneration, Shandong University, Jinan, China; 5 Shandong Provincial Clinical Research Center for Oral Diseases, Shandong University, Jinan, China; 6 Clinical Medical School (Affiliated Hospital), Jining Medical University, Jining, China; 7 Department of Periodontology, Jinan Stomatological Hospital, Jinan, China

**Keywords:** diagnosis, meta-analysis, oral potentially malignant disorders, systematic review, VELscope

## Abstract

**Purpose:**

This study aimed to evaluate the diagnostic efficacy of VELscope in detecting oral potentially malignant disorders (OPMD).

**Methods:**

A systematic search was conducted across six English databases and two Chinese databases: Embase, Web of Science, PubMed, Scopus, Dentistry & Oral Sciences Source (EBSCO), CINAHL (EBSCO), China National Knowledge Infrastructure (CNKI), and Chinese biomedical literature service system (SinoMed). Prospective and cross-sectional studies using VELscope to diagnose OPMD were included. Study quality was assessed using QUADAS-2. The meta-analysis evaluated VELscope's sensitivity, specificity, positive likelihood ratio, negative likelihood ratio, and the summary receiver operating characteristic (SROC) curve. Heterogeneity was assessed via Deeks’ funnel plot asymmetry test, bivariate boxplot, and meta-regression analysis to identify sources of variation.

**Results:**

A total of 2054 studies were retrieved, of which 24 were included in the meta-analysis. The sensitivity of VELscope was 84% (95% CI, 0.78–0.89; I^2^ = 78.0%), specificity was 45% (95% CI, 0.33–0.59; I^2^ = 93.8%), and the positive likelihood ratio was 1.55 (95% CI, 1.22–1.96; I^2^ = 86.9%). The area under the SROC curve was 0.78 (95% CI, 0.74–0.81). Subgroup analysis indicated that SCIE indexing, comparative study design, and high risk of bias influenced the sensitivity of VELscope.

**Conclusion:**

The VELscope demonstrates high sensitivity but limited specificity in diagnosing OPMD. Although it is unreasonable to rely solely on VELscope for a definitive diagnosis, it can provide valuable adjunctive support for detecting OPMD in settings such as large-scale screenings, community oral examinations, and resource-limited environments.

**Systematic Review Registration:**

PROSPERO (CRD42025364593).

## Introduction

Oral potentially malignant disorders (OPMD) represent a group of conditions in the oral cavity that carry a risk of progression to cancer (1). Typical forms of OPMD include leukoplakia, proliferative verrucous leukoplakia, erythroplakia, oral submucous fibrosis, and oral lichen planus (2, 3). The overall malignant transformation rate of OPMD is 7.9% (4). Currently, no effective methods are available to prevent their progression to oral squamous cell carcinoma (5). Therefore, the timely and accurate detection of OPMD is of great significance for providing adequate treatment, educating patients about associated risks, and preventing oral cancer (3, 6).

The conventional screening methods for OPMD involve visual examination under direct light and oral palpation, followed by biopsy and histopathological examination for definitive diagnosis (7). Traditional oral examinations have been shown to be effective in resource-limited settings; however, visual inspection and palpation of the oral cavity depend on the clinician's judgment and experience (8). Additionally, biopsy and histopathological examination are invasive and technique-sensitive procedures that require additional laboratory work and incur extra costs, making them unsuitable for routine clinical screening (1). Several non-invasive imaging techniques have recently been developed and employed as adjunctive tools for the screening of OPMD and oral cancer, including autofluorescence, optical coherence tomography, and clinical photography (7). In particular, the use of VELscope provides a rapid and non-invasive solution for OPMD screening (6, 7).

The VELscope is an autofluorescence-based imaging device that utilizes blue light excitation within the 400–460 nm range to activate endogenous fluorophores in oral mucosal tissues (7). It detects premalignant or early cancerous lesions by highlighting differences in fluorescence: healthy mucosa appears green, while abnormal regions show reduced or absent emission (7, 9). The benefits of VELscope include non-invasiveness, operational simplicity, improved early detection rates, and support in defining lesion boundaries and monitoring changes (3, 9). Despite widespread clinical use, the reported diagnostic accuracy and specificity vary considerably across studies partly due to operator experience and environmental factors, limiting its reliability in routine practice (3, 7).

While recent reviews have assessed the efficacy of autofluorescence in diagnosing OPMD, their performance varies considerably across different technologies (10, 11). Furthermore, the general diagnostic value of autofluorescence does not directly reflect the specific performance of the VELscope device. Given the widespread clinical use of VELscope and the current lack of a dedicated meta-analysis focusing specifically on its diagnostic performance, we conducted this study to evaluate the diagnostic efficacy of VELscope for OPMD.

## Materials and methods

### Protocol and registration

This study was performed following the Preferred Reporting Items for Systematic Reviews and Meta-Analyses for Diagnostic Test Accuracy (PRISMA-DTA) Studies (12), and the PRISMA-DTA checklist is provided in Appendix Table 1. The study protocol was registered on the International Prospective Register of Systematic Reviews. The protocol was registered in PROSPERO (CRD42025364593).

**Table 1:** Characteristics of the included studies.

Auther	Year	Country or region	Sample size（n）	Male/female（n）	Age (years)	Types of lesions evaluated	Comparison group	Conclusion (positive/negative)
Adil (15)	2017	India	56	75/15	NR	Speckled leukoplakia, homogeneous leukoplakia, verrucous leukoplakia, and erythroplakia	Toluidine blue staining	Positive
Amirchagh (16)	2018	Iran	30	28/26	52.3 ± 14.8	Leukoplakia, erythroplakia, and oral submucosal fibrosis, and premalignant conditions	No	Positive
Awan (17)	2011	United Kingdom	126	70/56	NR	Oral leukoplakia/erythroplakia, oral lichen planus, chronic hyperplastic candidiasis, rest frictional keratosis, and oral submucous fibrosis	No	Positive
Cânjău (18)	2018	Romania	18	12/6	61.4 ± 5.7	Erythematous, ulcerative	No	Positive
Chiang (19)	2019	Taiwan	116	110/16	NR	Leukoplakia, erythroplakia, erythroleukoplakia, verrucous hyperplasia, submucosal fibrosis, lichen planus, and suspected oral cancer	No	Positive
Farah (20)	2012	Australia	112	46/66	58.6 ± 12.4	Homogenous leukoplakia/keratosis, nonhomogenous leukoplakia/suspicious for malignancy, lesions with lichenoid features, others	Clinical examination	Negative
Ganga (21)	2017	India	200	NR	NR	Oral submucous fibrosis, leukoplakia, oral lichen planus, pyogenic granuloma, etc.	No	Negative
Gao (22)	2025	China	34	17/17	NR	Infiltrative, exophytic, and ulcerative	No	Positive
Hang (23)	2019	China	28	10/18	NR	Oral leukoplakia, oral lichen planus, erythroplakia, oral submucous fibrosis, chronic discoid lupus erythematosus	No	Positive
Koch (24)	2011	Germany	78	46/32	61.7	Red features like erythroplakia or erythroleukoplakia, white/hyperkeratotic feature, ulcerous, speckled aspect	No	Positive
Leuci (25)	2020	Italy	35	17/18	58.5	Leukoplakia, erythroplakia, oral lichen planus, lichenoid lesions, unspecified keratotic lesions	No	Positive
Mehrotra (26)	2010	India	156	140/16	41	NR	ViziLite	Negative
Paderni (27)	2011	Italy	147	79/96	60.38 ± 12.26	Oral leukoplakia, oral lichen planus, oral lichenoid lesions, verrucous proliferative leukoplakia, leuko-erythroplakia	No	Positive
Petruzzi (28)	2014	India	49	27/22	56.7	NR	Toluidine blue staining	Positive
Scheer (29)	2011	Germany	64	39/25	59.8	Red, white, mixed lesion, and ulcus	No	Positive
Sharma (30)	2022	India	250	NR	50	Leukoplakia, Erythroplakia, lichen planus or pemphigus vulgaris, Verrucous hyperplasia, etc.	No	Positive
Shi (31)	2019	China	517	238/279	51.9	Oral white, red, mixed red and white lesions	No	Positive
Tatapudi (32)	2025	India	30	NR	NR	Oral leukoplakia, oral lichen planus or oral submucous fibrosis	Toluidine blue staining	Positive
Vibhute (33)	2021	India	30	19/11	53.86	Leukoplakia, erythroplakia	No	Positive
Wang (34)	2022	China	54	22/33	58 ± 12.6	Oral leukoplakia or oral lichen planus	No	Positive
Wu (35)	2020	China	34	15/19	45.88 ± 7.61	Oral lichen planus, oral leukoplakia, oral erythroplakia, oral submucous fibrosis, etc.	No	Positive
Yamamoto (36)	2017	Japan	79	31/31	59.6	Suspected of epithelial dysplasia	Iodine staining	Positive
Yeladandi (37)	2024	India	40	26/14	47.6 ± 12.6	NR	No	Positive
Zhu (38)	2025	China	80	NR	NR	Suspected dysplasia	No	Positive

NR, not report; ViziLite, an autofluorescenc detection device similar to VELscope. Age is presented as mean ± SD in years.

### Inclusion and exclusion criteria

The inclusion criteria were developed according to the PICOS (population, intervention, comparison, outcomes, and study design) principle (12). *Population (P)*: patients with OPMD. Intervention (I): Use of VELscope for diagnosis of OPMD with pathological diagnosis as the gold standard. *Comparison (C)*: studies evaluating the diagnostic efficacy of VELscope or comparing its diagnostic efficacy with other methods (e.g., chemical staining). *Outcomes (O)*: studies reporting or allowing calculation of true positive (TP), false positive (FP), true negative (TN), and false negative (FN) values. *Study design (S)*: prospective or cross-sectional studies. OPMD were defined as oral mucosal abnormalities that are associated with a statistically increased risk of developing oral cancer, including conditions such as leukoplakia, erythroplakia, oral submucosal fibrosis, lichen planus, and discoid lupus erythematosus (6, 7).

The following studies were excluded: (1) studies unrelated to the topic, such as evaluations of oral squamous cell carcinoma (OSCC) recurrence, determination of intraoperative margins for OSCC, or changes before and after treatment for OPMD; (2) animal studies; (3) *in vitro* studies; (4) editorials, letters, personal opinions, conference abstracts; (5) duplicate publications.

### Literature search

A comprehensive search was conducted across electronic databases including PubMed, Embase, Web of Science, Dentistry & Oral Sciences Source (EBSCO), CINAHL (EBSCO), SinoMed, and CNKI. The search was conducted on October 8, 2025, and no restrictions were imposed on study type, time period, or publication status. Search terms encompassed ‘Autofluorescence,’ ‘VELscope,’ and were combined with ‘Oral potentially malignant disorders’. Using the PubMed search formula (Appendix Table 2) as a benchmark, the search strategy was adapted according to the characteristics of each database. Additionally, researchers manually reviewed reference lists of relevant studies and reviews.

**Table 2:** Main outcomes of included studies.

Number	Auther	Year	Number (n)	TP	FP	TN	FN	Sensitivity (%)	Specificity (%)	PPV (%)	NPV (%)
1	Adil (15)	2017	56	39	1	4	12	76.5	80.0	97.5	25.0
2	Amirchagh (16)	2018	30	10	16	2	2	83.3	11.1	38.5	50.0
3	Awan (17)	2011	126	61	44	12	9	87.1	21.4	58.1	57.1
4	Cânjău (18)	2018	18	16	0	1	1	94.1	100.0	100.0	50.0
5	Chiang (19)	2019	116	53	31	17	15	77.9	35.4	63.1	53.1
6	Farah (20)	2012	118	8	34	57	19	29.6	62.6	19.0	75.0
7	Ganga (21)	2017	200	19	59	116	6	76.0	66.3	24.4	95.1
8	Gao (22)	2025	34	24	3	1	6	80.0	25.0	88.9	14.3
9	Hang (23)	2019	28	17	5	3	3	85.0	37.5	77.3	50.0
10	Koch (24)	2011	78	28	41	7	2	93.3	14.6	40.6	77.8
11	Leuci (25)	2020	35	13	2	18	2	86.7	90.0	86.7	90.0
12	Mehrotra (26)	2010	156	6	88	56	6	50.0	38.9	6.4	90.3
13	Paderni (27)	2011	147	12	10	121	4	75.0	92.4	54.5	96.8
14	Petruzzi (28)	2014	56	13	19	20	4	76.5	51.3	40.6	83.3
15	Scheer (29)	2011	64	12	10	42	0	100.0	80.8	54.5	100.0
16	Sharma (30)	2022	250	78	36	124	12	86.7	77.5	68.4	91.2
17	Shi (31)	2019	517	37	315	165	0	100.0	34.4	10.5	100.0
18	Tatapudi (32)	2025	30	19	6	3	2	90.5	33.3	76.0	60.0
19	Vibhute (33)	2021	30	21	3	1	5	80.8	25.0	87.5	16.7
20	Wang (34)	2022	59	23	14	14	8	74.2	50.0	62.2	63.6
21	Wu (35)	2020	34	18	12	3	1	94.7	20.0	60.0	75.0
22	Yamamoto (36)	2017	79	55	11	4	9	85.9	26.7	83.3	30.8
23	Yeladandi (37)	2024	40	24	7	6	3	88.9	46.2	77.4	66.7
24	Zhu (38)	2025	80	15	58	6	1	93.8	9.4	20.5	85.7

TP, true positive; FP, false positive; TN, true negative; FN, false negative; PPV, positive predictive value; NPV, negative predictive value.

### Study selection

All retrieved studies were imported into NoteExpress (version 4.2) to eliminate duplicate records. Two researchers independently screened the literature. Initial screening was conducted based on titles and abstracts, followed by full-text review to determine final inclusion. Disagreements between researchers were resolved through discussion; where consensus could not be reached, a third researcher was consulted.

### Data extraction

Two researchers independently extracted data using specially designed spreadsheets. For each included study, the following data were extracted: authors, year of publication, study location, study design (comparison group), lesion types of OPMD, participant characteristics (age, gender), study results, and conclusions. The primary outcomes extracted from each study comprised the values for TP, FP, TN, and FN. Additionally, information on sensitivity, specificity, positive predictive value, and negative predictive value was extracted from each study. Where these values were not directly reported in the original literature, they were calculated from the available data. A third researcher was responsible for consolidating the data, resolving any discrepancies between researchers, and reaching consensus.

### Quality assessment

The methodological quality of all included studies was assessed using the Quality Assessment of Diagnostic Accuracy Studies-2 tool (QUADAS-2) within Review Manager software (version 5.3) (13). The risk of bias and clinical applicability of the included studies were assessed across four domains: patient selection, diagnostic method under evaluation, gold standard, and case flow and timing. The risk of bias in the included studies was categorised as ‘high risk’, ‘unclear’, or ‘low risk’, while clinical applicability was rated as ‘high applicability’, ‘uncertain applicability’, or ‘low applicability’.

### Statistical analyses

The pooled sensitivity and specificity of VELscope were determined using a random-effects model, with 95% confidence interval (CI) serving as effect size measures. The I^2^ statistic was used to assess heterogeneity between studies, with I^2^ > 50% indicating significant statistical heterogeneity. Spearman's correlation coefficient was used to assess the threshold effect between sensitivity and specificity. Potential publication bias was assessed using funnel plots and Egger's test. A summary receiver operating characteristic (SROC) curve was constructed, and the area under the curve (AUC) calculated (14). Furthermore, potential influencing factors were explored via meta-regression analysis (11). Statistical analyses were performed using Stata (version 16.0), with statistical significance set at P < 0.05.

## Results

### Literature search and study selection

**Figure 1:**
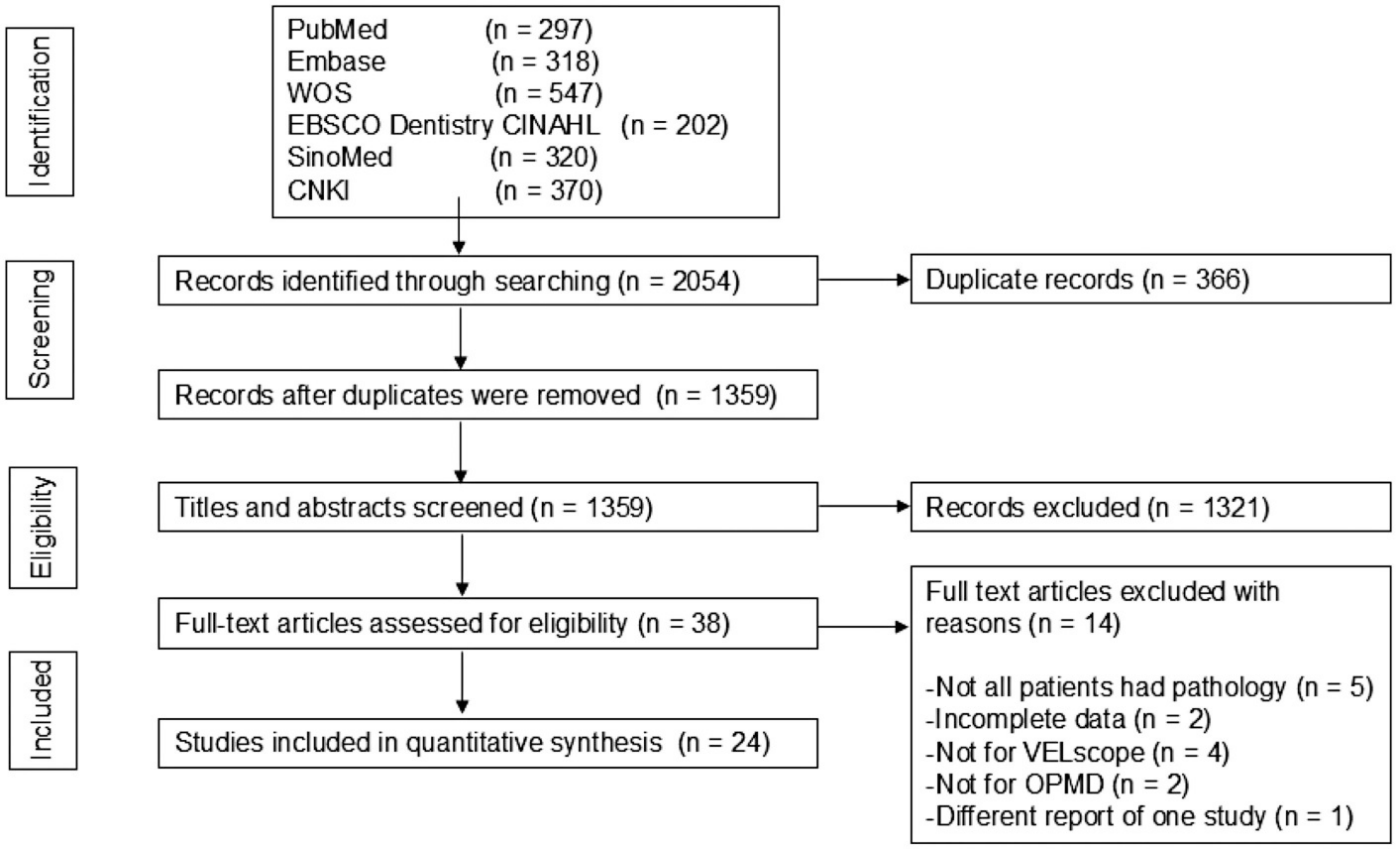
The PRISMA flowchart of literature searching and screening. The flow diagram illustrates the process of study identification, screening, eligibility assessment, and final inclusion according to the PRISMA guidelines. PRISMA, Preferred Reporting Items for Systematic Reviews and Meta Analyses; WOS, Web of Science; CNKI, China National Knowledge Infrastructure; SinoMed, Chinese biomedical literature service system.

This study retrieved a total of 2,054 publications, which were reduced to 1,359 after removing duplicates. After an initial screening based on titles and abstracts, 38 publications were selected. Following full-text review, 14 papers were excluded for the following reasons: not all patients underwent pathological examination (five papers), incomplete data (two papers), not evaluating for VELscope (four papers), studies not specifically addressing OPMD (two papers), and duplicate reporting of the same study (1 paper). The flowchart for literature retrieval and screening is presented below (Figure 1). Ultimately, 24 publications were included in this study.

### Characteristics and main outcomes of the included studies

Twenty-four eligible studies collectively enrolled 2,363 patients (Table 1), with the mean age of participants ranging from 40 to 60 years. Eighteen studies directly evaluated the diagnostic efficacy of VELscope, whilst six studies compared its performance against alternative methods: toluidine blue staining (three studies), iodine staining (one study), other autofluorescence detection devices (one study), and oral examination (one study). Among the included studies, 21 reported that VELscope aids in the diagnosis of OPMD, while three studies found no significant clinical benefit from its use. Since OPMD is a broad term encompassing a spectrum of diseases, there is heterogeneity in the specific lesion types included across the studies analyzed (Table 1). Some studies enrolled a limited range of lesion types, while others incorporated a more extensive variety. In addition, some studies did not report the specific lesion types involved.

The primary results from the included studies are presented in Table 2. The sensitivity reported in the included studies ranged from 30% to 100%, while the specificity varied more widely (9.4% to 100%). The reported positive predictive values ranged from 6.4% to 100%, and the negative predictive values ranged from 14.3% to 100%.

### Quality assessment

**Figure 2:**
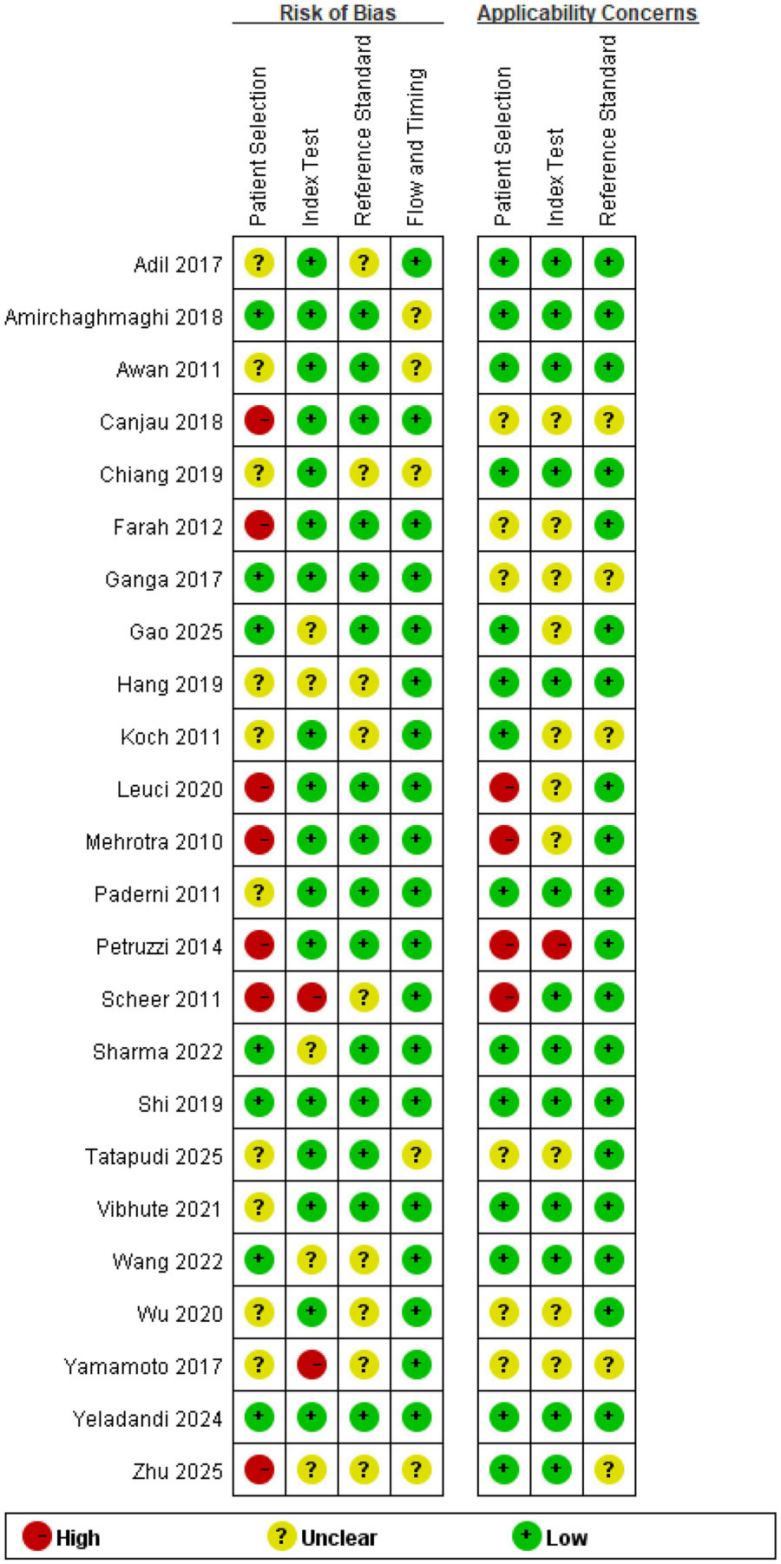
Risk of bias and applicability concerns graph basing on QUADAS-2 tool. Risk of bias was assessed across four domains: patient selection, index test, reference standard, and flow and timing. Each circle represents the judgment for an individual study in each domain. Green indicates low risk, yellow indicates unclear risk, and red indicates high risk of bias or applicability concern.

The results of the risk of bias assessment for included studies using the QUADAS-2 are shown in Figure 2. Among the 24 studies included in this systematic review, eight studies were rated as “high risk”, three studies were rated as “low risk”, and the remaining 13 studies were rated as “unclear risk”. The primary source of high risk was “Patient selection,” accounting for approximately 87.5%, while the “Reference standard” did not contribute to high risk. Among the included studies, 11 studies showed high clinical applicability, while three studies had unclear applicability concerns.

### Meta-analysis

**Figure 3:**
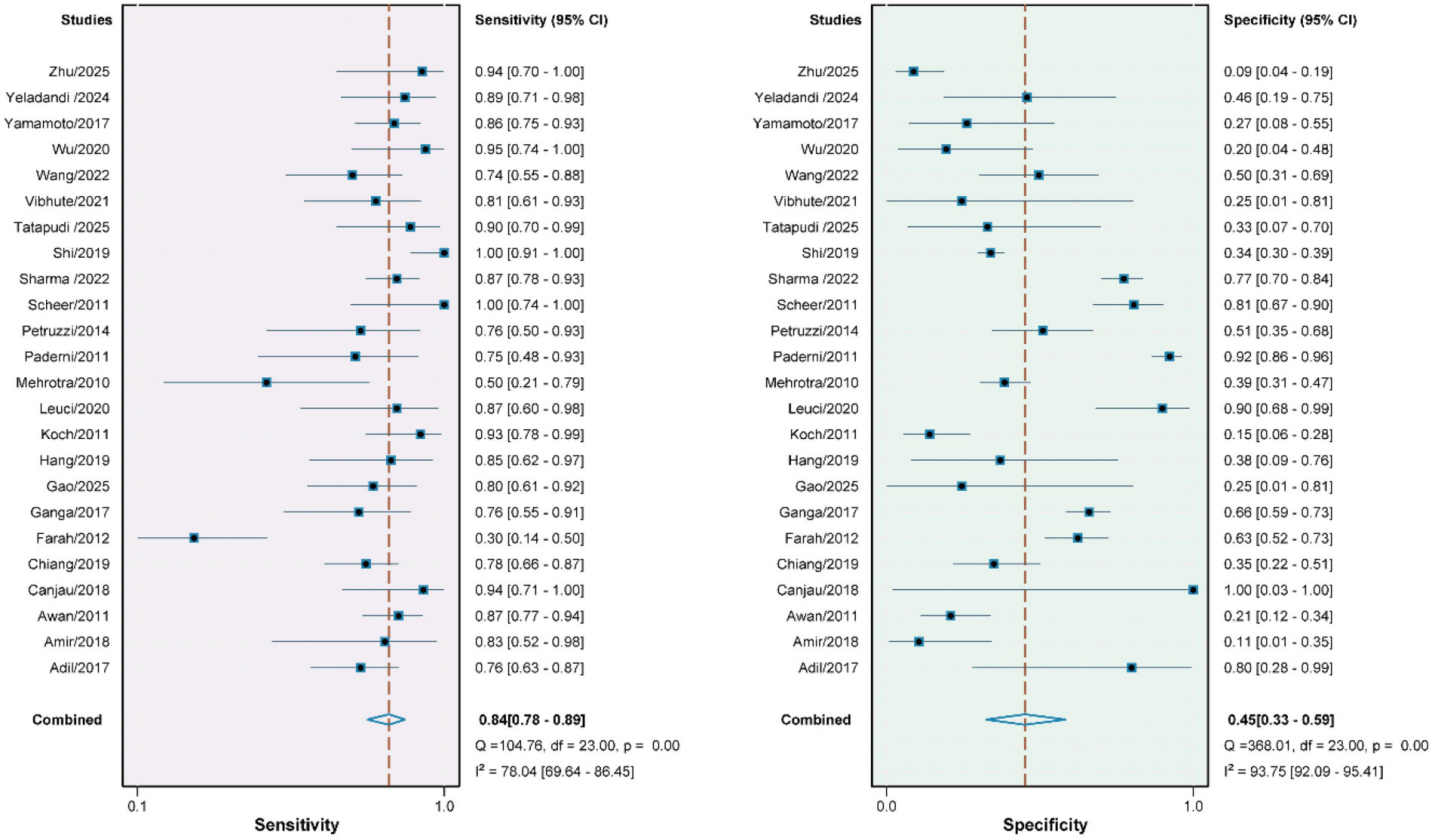
Forest plot showing the sensitivity (left) and specificity (right) of VELscope in diagnosing OPMD. Each square represents the point estimate of an individual study, and the horizontal line indicates the 95% confidence interval. The dashed vertical line marks the pooled estimate, while the diamond at the bottom represents the overall combined result.

The Spearman rank correlation coefficient was 0.326 (*P* = 0.119), indicating no threshold effect in this meta-analysis and allowing for the pooling of study results. The pooled sensitivity and specificity of VELscope in discriminating benign from malignant lesions were 0.84 (95% CI, 0.78–0.89) and 0.45 (95% CI, 0.33–0.59), respectively (Figure 3). The pooled positive likelihood ratio was 1.55 (95% CI, 1.22–1.96; I^2^ = 86.9%) (Appendix Fig. 2). The pooled diagnostic score was 1.51 (95% CI, 0.92–2.10; I^2^ = 74.6%) (Appendix Fig. 3).

**Figure 4:**
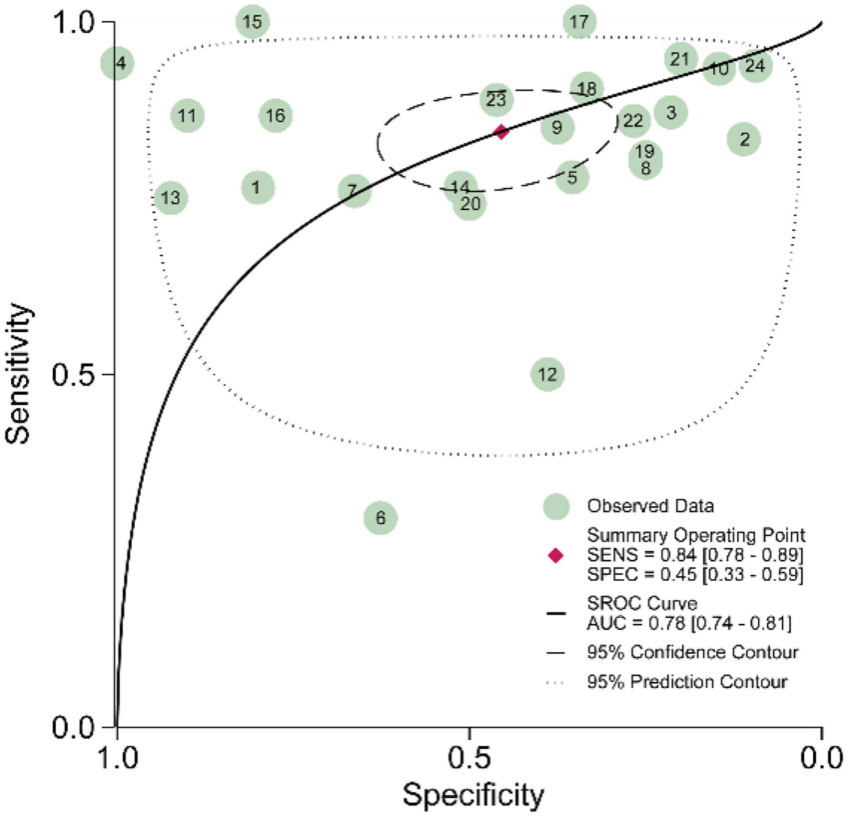
Receiver operating characteristic (SROC) curve of the diagnostic performance of VELscope. The plot shows the paired sensitivity and specificity estimates from the included studies, with each circle representing one study. The pooled estimate is shown together with the 95% confidence region and 95% prediction region, illustrating the overall diagnostic accuracy of VELscope and the heterogeneity among studies.

An AUC value greater than 0.5 indicates that a method is valuable, and a higher AUC value, reflects greater diagnostic accuracy. In this meta-analysis, the estimated AUC for VELscope was 0.78 (95% CI, 0.74–0.81) (Figure 4).

### Heterogeneity analysis and meta regression

**Figure 5:**
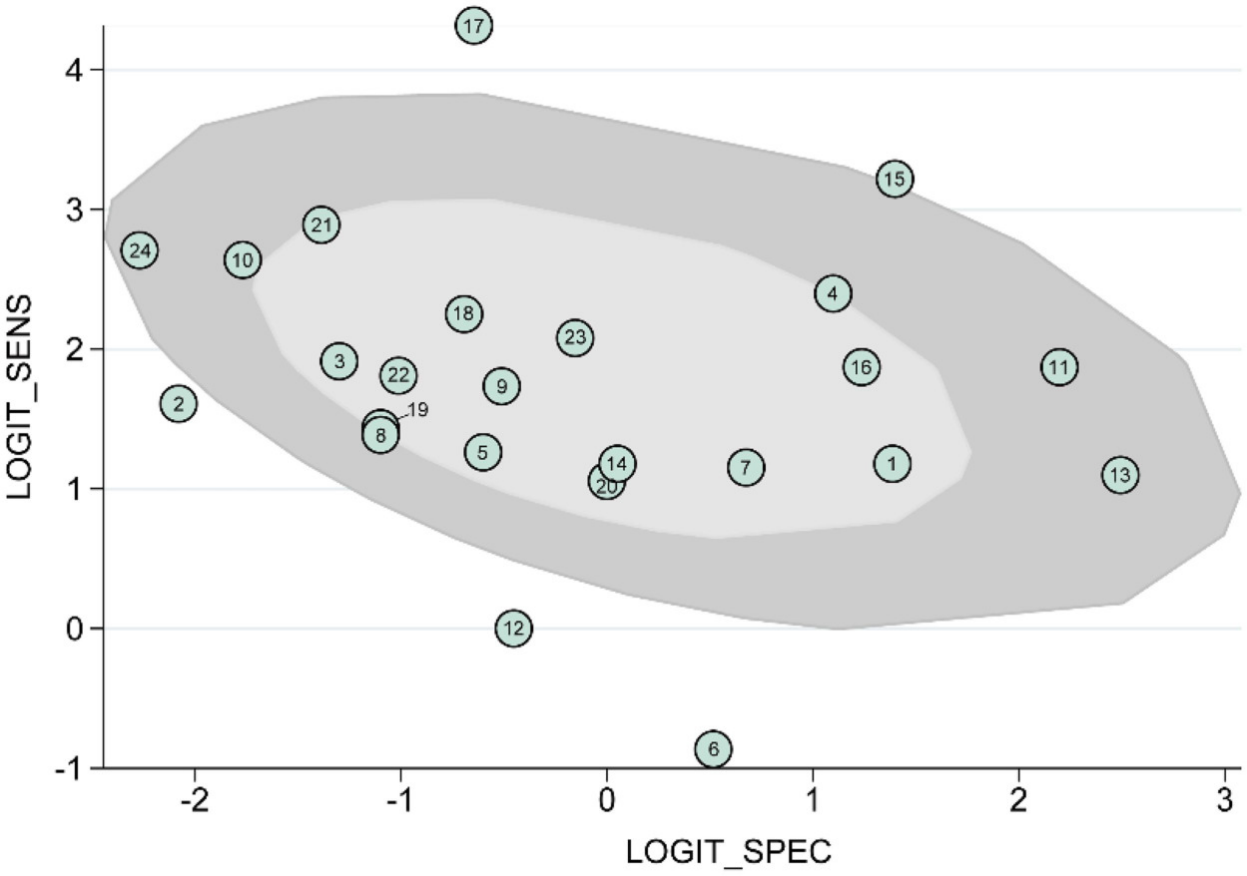
Bivariate boxplot evaluating the heterogeneity. Each circle represents an individual study included in the meta-analysis. The distribution of the studies within the central region and surrounding contours reflects the between-study heterogeneity in sensitivity and specificity.

The I^2^ values for the combined sensitivity and specificity were 78.0% and 93.7%, respectively. Given the high heterogeneity observed in pooled statistics, we analyzed the sources of heterogeneity using bivariate boxplots of sensitivity vs. specificity (Figure 5). The inner ellipse in the figure shows studies within the median distribution range, while the outer ellipse indicates the 95% confidence interval. The figure indicates that heterogeneity may originate from six studies (16, 20, 26, 29, 31, 38). Moreover, as showing in Appendix Fig. 1, the Deeks’ funnel plot asymmetry test indicated no significant publication bias (*P* = 0.32).

**Figure 6:**
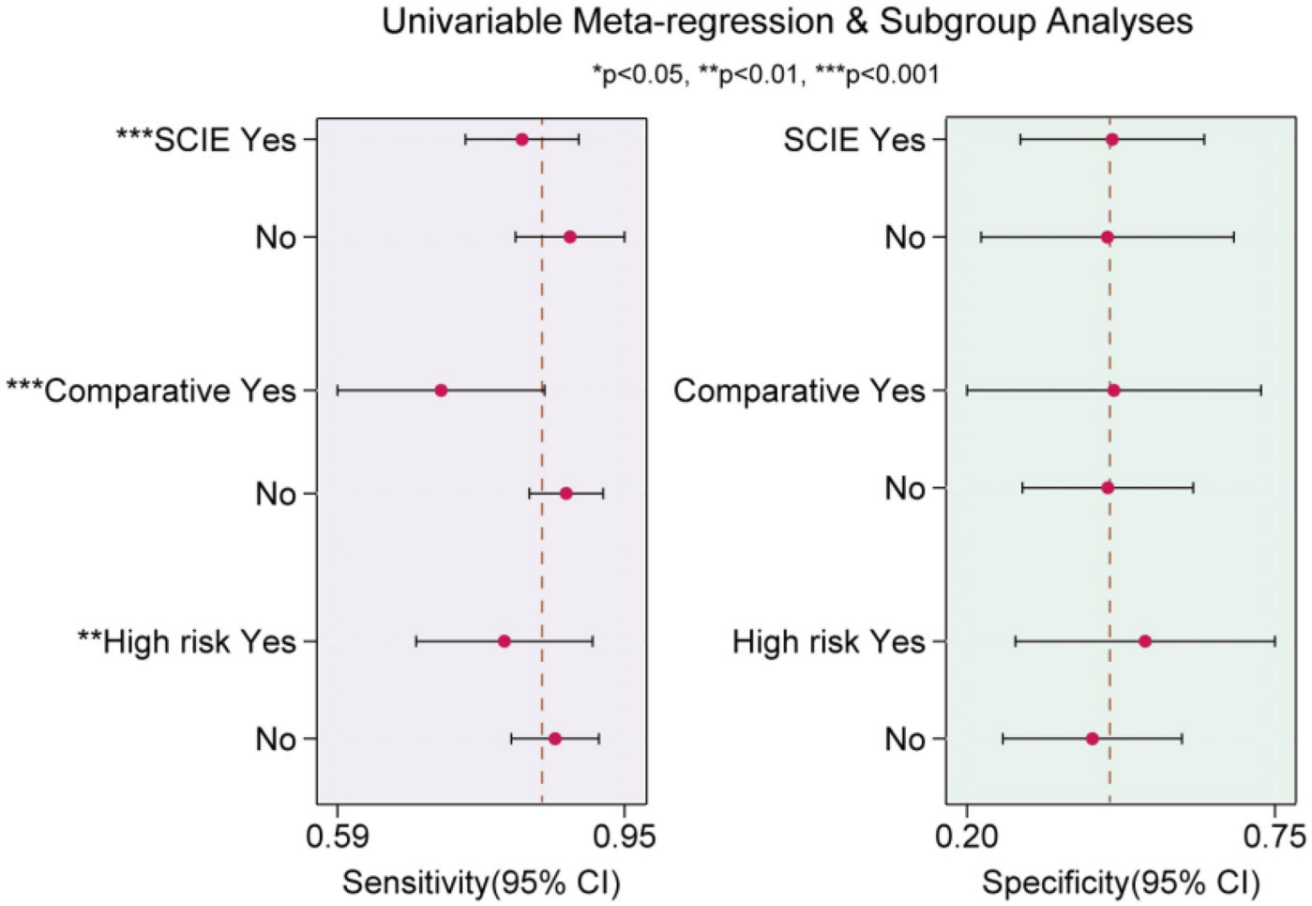
Meta-regression and subgroup analyses based on sensitivity (left) and specificity (right). Subgroup analyses were performed based on study quality (SCIE indexed), study design (comparative study), and risk level (high risk of bias). The plots show pooled sensitivity and specificity with their 95% confidence intervals for each subgroup. The dashed vertical line indicates the overall pooled estimate.

To further investigate the sources of heterogeneity, we conducted a logistic meta-regression analysis (Figure 6). The results indicated that heterogeneity in sensitivity may be associated with whether studies were SCIE indexed, comparative study, or high-risk study.

## Discussion

This systematic review and meta-analysis synthesized data from 24 clinical studies to evaluate the diagnostic accuracy of VELscope for OPMD. Our findings showed that VELscope demonstrated a pooled sensitivity of 84% (95% CI, 0.78–0.89; I^2^ = 78.0%) and a specificity of 45% (95% CI, 0.33–0.59; I^2^ = 93.8%). Several previous studies have reported similar finding, although the reported diagnostic performance varies across studies. One study reported that autofluorescence more accurately identified premalignant and malignant lesions (sensitivity = 0.86; 95% CI, 0.77–0.91) compared with chemiluminescence (sensitivity = 0.67; 95% CI, 0.38–0.87) and conventional visual examination (sensitivity = 0.63; 95% CI, 0.45–0.78) (39). Another study to examine diagnostic applicability of autofluorescence imaging in early detection of OPMD found that VELscope was the most common used equipment, and VELscope had an overall sensitivity and specificity of 74% and 57% respectively (40). More recently, another study reported that AI-assisted screening achieved a sensitivity of 89.9% (95% CI, 0.866–0.925; I^2^ = 81%) and a specificity of 89.2% (95% CI, 0.851–0.922; I^2^ = 79%) (6). Compared with these previous studies, our study distinctively focuses solely on the VELscope device, which reduces heterogeneity across included studies and provides more direct, applicable evidence for its clinical use. Furthermore, our comprehensive literature search allowed inclusion of a larger number of studies, and the application of rigorous statistical and subgroup analyses provides more robust evidence.

OPMD are a group of conditions that do not inevitably progress to oral cancer but are associated with a significantly increased risk of malignant transformation (41). Screening and early diagnosis of OPMD to detect high-risk lesions are clinically essential for intervening in cancer progression (5). However, the diversity of anatomical/morphological structures and their composition pose challenges in developing accurate screening and diagnostic methods. Over decades, numerous techniques based on dyes or spontaneous fluorescence and spectral patterns have been explored (3). This review examined and analyzes the autofluorescence imaging technology of VELscope, commonly used in clinical practice, with a primary focus on its diagnostic efficacy for OPMD.

Among the 24 included studies, 18 exclusively analyzed the diagnostic efficacy of VELscope, while six compared its efficacy with other diagnostic methods. These studies spanned from 2010 to 2025 across 10 countries or regions, indicating sustained global interest in VELscope for diagnosing OPMD. Among these 24 studies, eight were assessed as “high risk,” and 13 as “unclear risk”. The primary source of high risk was “patient selection,” accounting for approximately 87.5%, with the other two sources being the index test and flow and timing. Under the patient selection category, the primary concerns were the absence of continuous enrollment (18, 20, 28) and unreasonable exclusion criteria (20, 26, 28, 38). This was related to the inclusion criteria applied in this study. We required all subjects to undergo pathological testing as the gold standard, thereby avoiding heterogeneity in the “reference standard.” Furthermore, this highlights the need for careful attention to patient enrollment methods and the establishment of reasonable exclusion criteria in future similar studies.

Sensitivity and specificity are key indicators for evaluating diagnostic performance (5). Among all included studies, 21 supported the diagnostic efficacy of VELscope for OPMD, particularly its utility as an adjunctive screening tool. These studies demonstrated that VELscope exhibited high sensitivity ranging from 77.9% to 100%, consistent with findings from previous meta-analyses (9, 42). However, three studies concluded that VELscope had limited diagnostic value (20, 21, 26), reporting sensitivities of 29.6%, 50%, and 76%, respectively. These sensitivities were among the lowest across all included studies. This discrepancy may be attributed to the comparative design of these three studies, as the subsequent meta-regression analysis indicated that comparative studies tend to underestimate VELscope's sensitivity (Figure 6). For example, Farah et al.'s study reported the lowest sensitivity of 29.6% among all studies (20). This discrepancy likely stems from their comparative design, which evaluated VELscope against conventional clinical examination. Consequently, the VELscope group did not undergo routine preliminary clinical assessment, whereas studies solely evaluating VELscope efficacy typically performed clinical examinations before VELscope examination. This difference explains the lower sensitivity observed in Farah et al.'s study. This suggests that comparative studies should be analyzed separately when investigating VELscope's diagnostic efficacy. Subsequent meta-analyses have similarly recommended separating purely diagnostic studies from comparative studies for analysis, or conducting subgroup analyses.

Meta-analyses can provide a higher level of evidence (43). In our study, the pooled sensitivity and specificity of VELscope were 0.84 (95% CI 0.78–0.89) and 0.45 (95% CI 0.33–0.59), respectively. This indicates that VELscope is a useful screening tool for timely detection of OPMD, but it is not suitable for direct clinical diagnosis due to its relatively high false-positive rate. This finding aligns with the results of the majority of included studies. VELscope's role in auxiliary diagnosis offers distinct advantages during large-scale screening for OPMD and oral cancer, particularly for general practitioners. This maximizes the reduction of missed diagnoses. Concurrently, the use of VELscope helps reduce unnecessary pathological examinations, alleviating patients’ financial burdens—a critical benefit in underdeveloped regions or areas with limited medical resources.

It is noteworthy that significant heterogeneity was observed for both sensitivity (I^2^ = 78.04, *P* = 0.00) and specificity (I^2^ = 93.75, *P* = 0.00). We conducted a detailed analysis of the sources of heterogeneity (13, 43). First, Deeks’ funnel plot asymmetry test indicated that heterogeneity did not originate from publication bias. Results from the box plot indicated that heterogeneity primarily stemmed from six studies (Figure 5). Meta-regression analysis (11) further confirmed that heterogeneity in sensitivity may be associated with whether the paper was SCIE indexed, comparative study, or high-risk of bias. SCIE-indexed papers and high-risk studies tended to overestimate VELscope diagnostic sensitivity, while comparative studies may have underestimated sensitivity. However, the meta-analysis did not identify any significant subgroup effects for specificity. This may be attributed to the insufficient specificity of VELscope, which further exacerbates heterogeneity across studies. Other potential sources of heterogeneity may include differences in the types of lesions, operator technique, and instrument parameter settings. However, due to insufficient data, we were unable to perform subgroup analyses based on these factors. We also recommend that future studies report such information in detail.

Our study has some limitations. First, existing research exhibits significant heterogeneity, necessitating cautious interpretation of current findings. Additionally, we did not include unpublished data or grey literature due to concerns over reliability and language barriers. Finally, methodological limitations were observed in most included studies, particularly regarding patient selection, index tests, reference standards, and flow and timing. These limitations are especially critical as they may compromise the reliability of current findings (13, 43). We hope that future higher-quality studies will address these shortcomings. In addition, it should be emphasized that the effective use of VELscope requires adequate training in its operation and in the interpretation of fluorescence images. Such training, ideally supported by a background in oral medicine or pathology, is crucial for optimizing its diagnostic performance in risk assessment of OPMD.

## Conclusions

This study demonstrates that VELscope has high sensitivity but limited specificity in diagnosing OPMD. In large-scale screening programs, routine dental examinations, and resource-limited settings, VELscope offers valuable adjunctive support for the detection of OPMD. Future studies should pay particular attention to patient inclusion and exclusion criteria to yield results with greater clinical guidance value. This research deepens our understanding of the VELscope's diagnostic utility for OPMD, ultimately guiding more refined, evidence-based clinical decision-making and subsequent investigations.

## Data Availability

The original contributions presented in the study are included in the article/Supplementary Material, further inquiries can be directed to the corresponding author.
