# Supplementary material for: Diagnostic efficacy of VELscope in oral potentially malignant disorders: a systematic review and meta-analysis
Source: Front Oral Health. 2026 Apr 17;7:1811909. doi: 10.3389/froh.2026.1811909 (PMC13132815; doi:10.3389/froh.2026.1811909)
Supplement: Supplementary file 1 [file Supplementaryfile1.docx]

## Appendix Tables

**Appendix Table 1.** **The PRISMA-DTA checklist.**


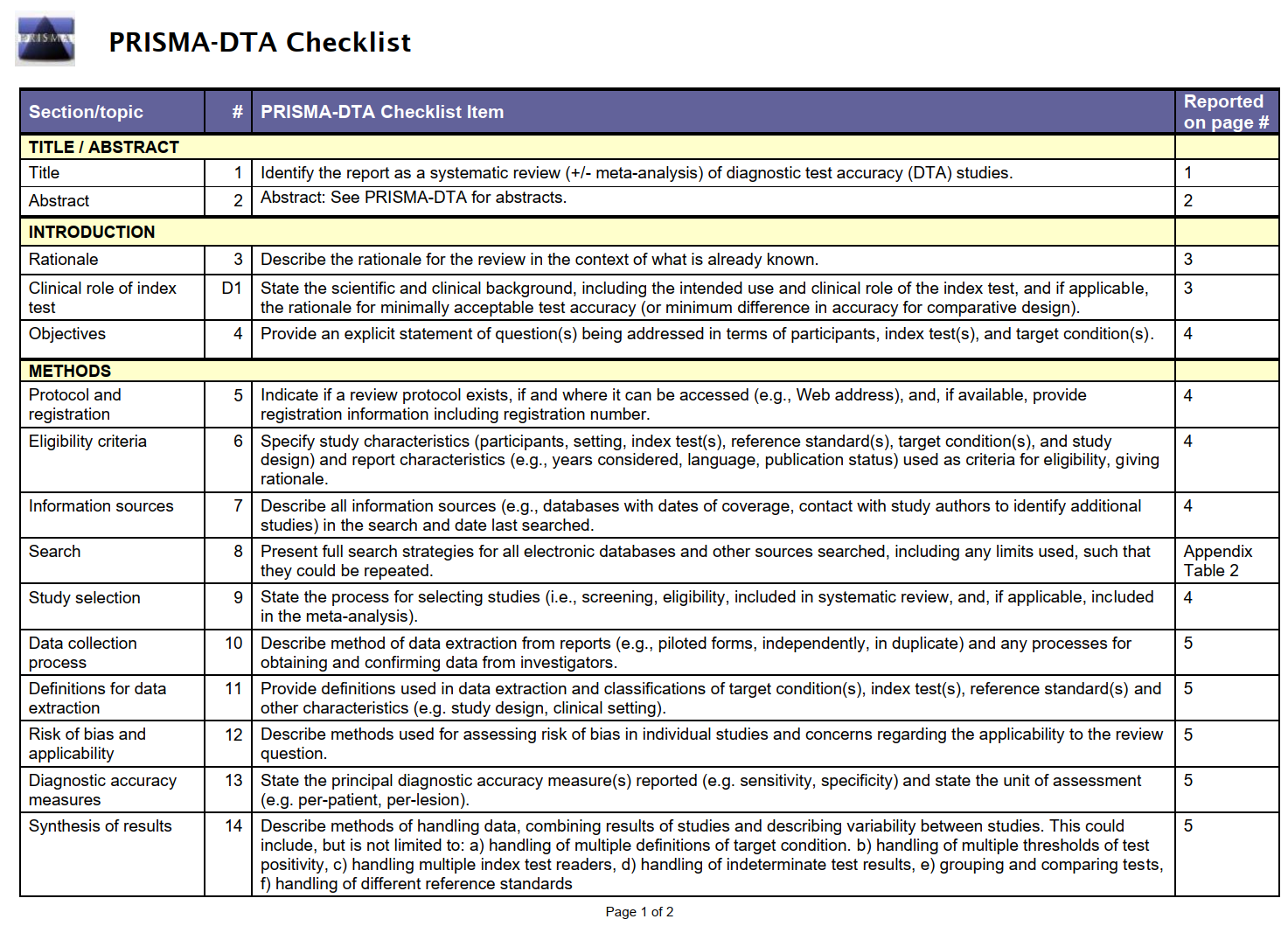


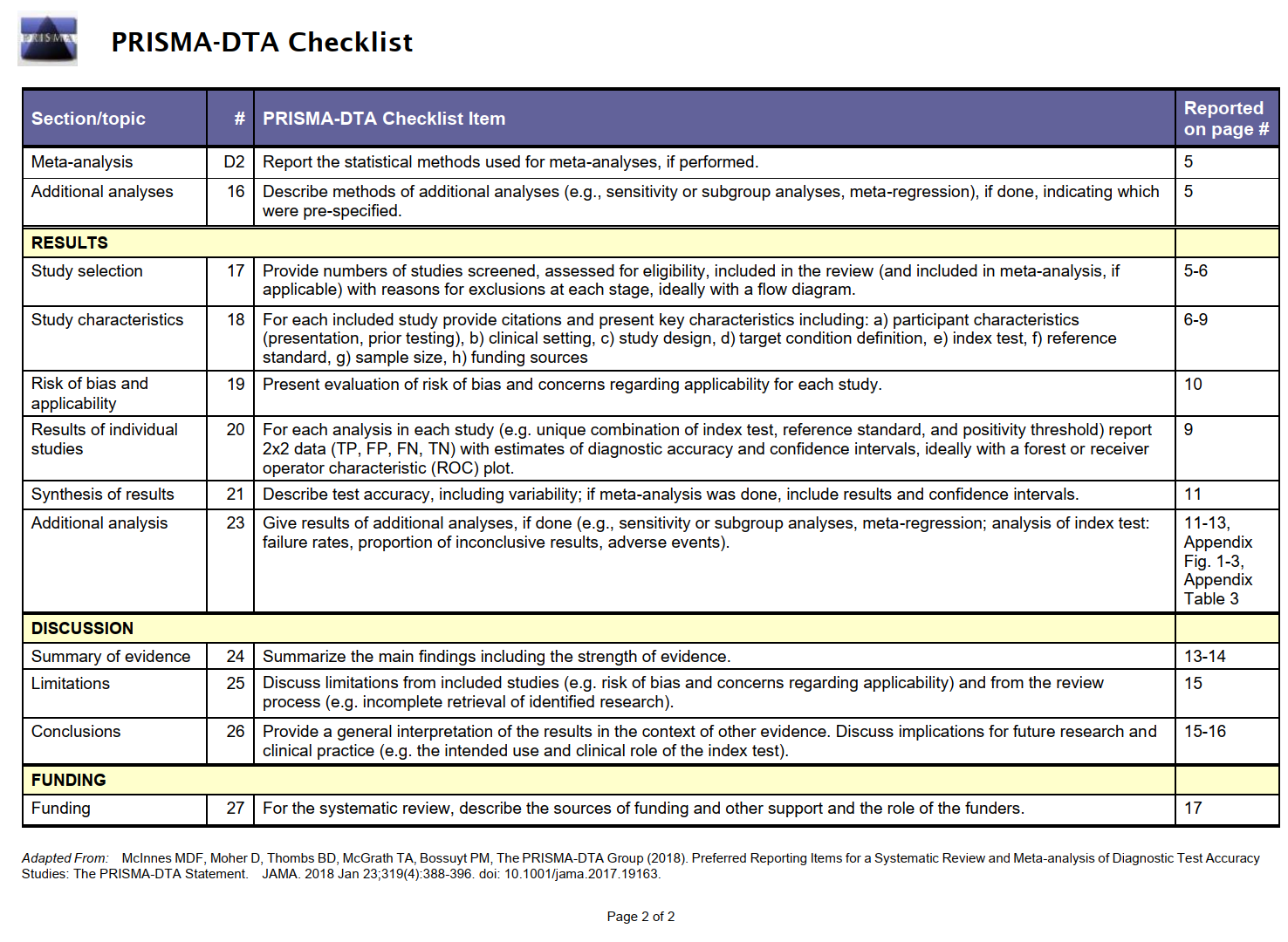


**Appendix Table 2. Search strategy of PubMed.**

| Search number | Query | Results |
| --- | --- | --- |
| 20 | #18 OR #19 | 239 |
| 19 | VELscope | 84 |
| 18 | #3 AND #17 | 185 |
| 17 | #4 OR #5 OR #6 OR #7 OR #8 OR #9 OR #10 OR #11 OR #12 OR #13 OR #14 OR #15 OR #16 | 34,803 |
| 16 | (Palatal lesions) AND (reverse smoking) | 18 |
| 15 | lip actinic keratosis | 73 |
| 14 | Syphilitic glossitis | 6 |
| 13 | Discoid lupus erythematosus | 4,264 |
| 12 | Lichen planus | 11,078 |
| 11 | Chronic candidiasis | 3,730 |
| 10 | Tobacco keratosis | 136 |
| 9 | Dyskeratosis congenita | 1,210 |
| 8 | Oral submucous fibrosis | 1,718 |
| 7 | Leukoplakia | 7,330 |
| 6 | Erythroleukoplakia | 68 |
| 5 | Erythroplakia | 797 |
| 4 | Oral potentially malignant disorders | 7,938 |
| 3 | #1 OR #2 | 71,575 |
| 2 | Optical Imaging[MeSH Terms] | 61,963 |
| 1 | Autofluorescence | 12,590 |

**Appendix Table 3. Meta-regression and subgroup analyse of elastosonography**

| **Covariate** | **Studies, *n*** | **Sensitivity (95%Cl)** | ***P-*Value** | **Specificity (95% CI)** | **P-Value** |
| --- | --- | --- | --- | --- | --- |
| SCIE |  |  | 0.00 |  | 0.85 |
| yes | 15 | 0.82 (0.75 - 0.89) |  | 0.46 (0.30 - 0.62) |  |
| no | 9 | 0.88 (0.81 - 0.95) |  | 0.45 (0.23 - 0.67) |  |
| Comparative |  |  | 0.00 |  | 0.87 |
| yes | 6 | 0.72 (0.59 - 0.85) |  | 0.46 (0.20 - 0.72) |  |
| no | 18 | 0.87 (0.83 - 0.92) |  | 0.45 (0.30 - 0.60) |  |
| High risk |  |  | 0.00 |  | 0.55 |
| yes | 8 | 0.80 (0.69 - 0.91) |  | 0.52 (0.29 - 0.75) |  |
| no | 16 | 0.86 (0.81 - 0.92) |  | 0.42 (0.27 - 0.58) |  |

## Appendix Figures


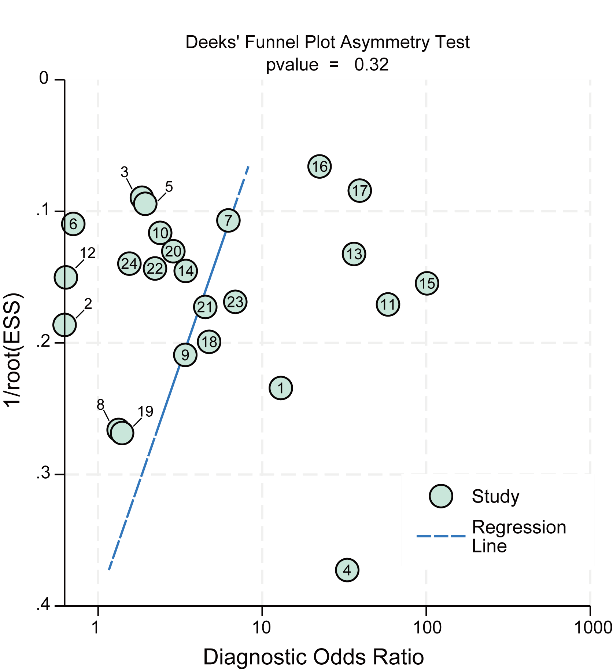


**Appendix Fig. 1. Deeks' funnel plot asymmetry test for publication bias.** Each circle represents an individual study included in the meta-analysis. The x-axis shows the diagnostic odds ratio (log scale), and the y-axis represents the inverse of the square root of the effective sample size (1/√ESS). The dashed blue line indicates the weighted regression line used in Deeks’ funnel plot asymmetry test. Symmetry of the distribution around the regression line suggests a low likelihood of publication bias (*P* = 0.32).


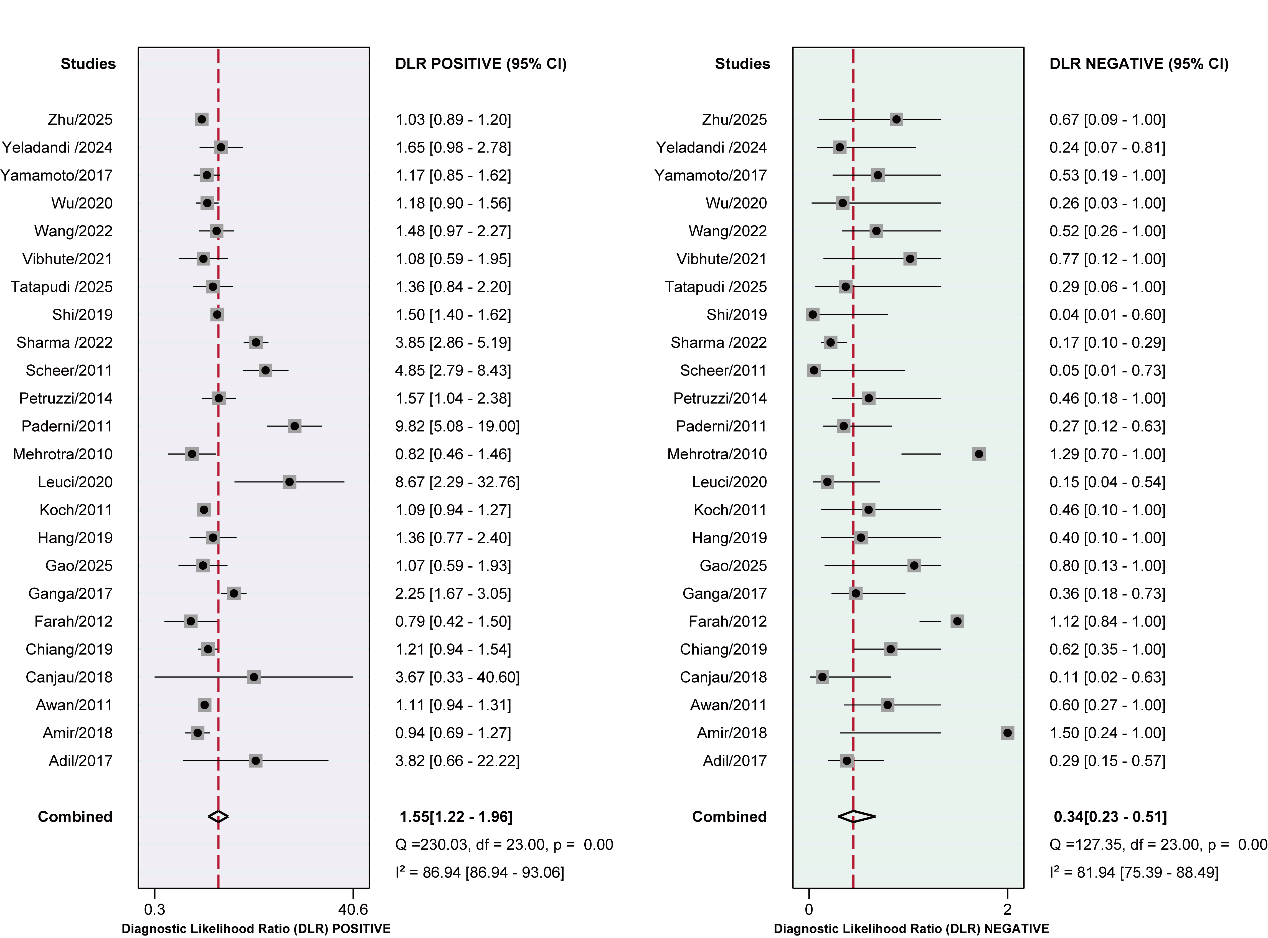


**Appendix Fig. 2. Forest plot showing the positive DLR and negative DLR of VELscope in diagnosing OPMD.** Each square represents the DLR estimate for an individual study, and the horizontal lines indicate the corresponding 95% CI. The dashed vertical line marks the pooled estimate, while the diamond at the bottom represents the overall combined result.


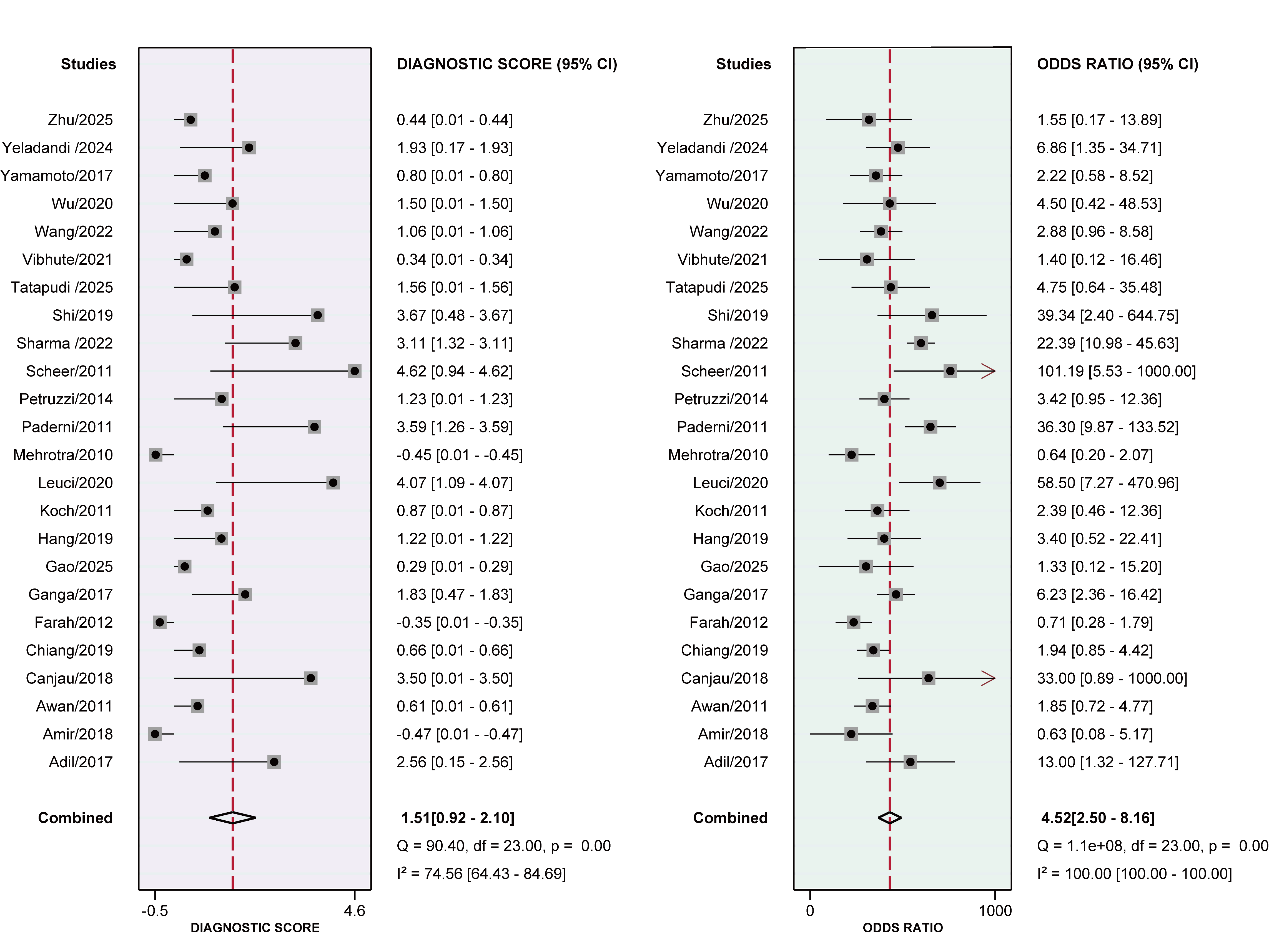


**Appendix Fig. 3. Forest plot showing the diagnostic score and odds ratio of VELscope in diagnosing OPMD.** Each square represents the estimate from an individual study, with horizontal lines indicating the corresponding 95% CI. The dashed vertical line marks the pooled estimate, while the diamond at the bottom represents the overall combined result.
